# Asymmetry Factors Shaping Regular and Irregular Bursting Rhythms in Central Pattern Generators

**DOI:** 10.3389/fncom.2017.00009

**Published:** 2017-02-16

**Authors:** Irene Elices, Pablo Varona

**Affiliations:** Grupo de Neurocomputación Biológica, Departamento de Ingeniería Informática, Escuela Politécnica Superior, Universidad Autónoma de MadridMadrid, Spain

**Keywords:** spiking-bursting activity, CPG, half-center oscillator, rhythm negotiation, rhythm regularization, closed-loop exploration

## Abstract

Central Pattern Generator (CPG) circuits are neural networks that generate rhythmic motor patterns. These circuits are typically built of half-center oscillator subcircuits with reciprocally inhibitory connections. Another common property in many CPGs is the remarkable rich spiking-bursting dynamics of their constituent cells, which balance robustness and flexibility to generate their joint coordinated rhythms. In this paper, we use conductance-based models and realistic connection topologies inspired by the crustacean pyloric CPG to address the study of asymmetry factors shaping CPG bursting rhythms. In particular, we assess the role of asymmetric maximal synaptic conductances, time constants and gap-junction connectivity to establish the regularity of half-center oscillator based CPGs. We map and characterize the synaptic parameter space that lead to regular and irregular bursting activity in these networks. The analysis indicates that asymmetric configurations display robust regular rhythms and that large regions of both regular and irregular but coordinated rhythms exist as a function of the asymmetry in the circuit. Our results show that asymmetry both in the maximal conductances and in the temporal dynamics of mutually inhibitory neurons can synergistically contribute to shape wide regimes of regular spiking-bursting activity in CPGs. Finally, we discuss how a closed-loop protocol driven by a regularity goal can be used to find and characterize regular regimes when there is not time to perform an exhaustive search, as in most experimental studies.

## 1. Introduction

Central Pattern Generators (CPGs) are neural networks that control muscle function by generating rhythmic patterns (Marder and Bucher, 2001; Grillner, 2003). Many CPGs are built with a non-open network architecture, i.e., a connection topology in which every neuron receives at least one synapse from another member of the CPG (Huerta et al., 2001). The building block in this type of connection architecture is often an oscillator circuit of reciprocally inhibitory neurons (Miller and Selverston, 1982; Selverston, 2010; Sakurai et al., 2014). Another highly relevant property in these neural networks is the presence of intrinsically irregular/chaotic neurons, which typically display rich slow-fast dynamics able to generate bursts of different duration periods, phases and spike temporal structures (Abarbanel et al., 1996; Elson et al., 1998). The reciprocal inhibitory connections between neurons lead to the regularization of the chaotic behavior when the neurons interact with each other in the circuit. The rich intrinsic dynamics provide flexibility and robustness for negotiating rhythms as a function of external inputs, producing the characteristic regular spiking-bursting activity that allows the CPG to control motor movements (Selverston et al., 2000). Most CPGs are typically studied in regular regimes where the activity of individual neurons can be directly related to specific rhythmic motor functions, such as walking, breathing, chewing, etc. However, irregular rhythms can also be observed in control or induced conditions (e.g., see Bartos et al., 1999; Thuma and Hooper, 2003; Nadim et al., 2011; Elices and Varona, 2015; Hooper et al., 2015). Central and sensory feedback transiently alter the CPG pattern which also results in observed irregularity.

Minimal circuits, such as half-center oscillators involving mutually inhibitory neurons are convenient networks to address CPG function. In particular, the concept of a half-center oscillator has been extensively used to study CPG rhythm generation, both in experimental, (e.g., see Miller and Selverston, 1982; Sharp et al., 1996; Yakovenko et al., 2005; Brookings et al., 2012; Sakurai et al., 2014), and theoretical works, (e.g., Nadim et al., 1995; Cymbalyuk et al., 2002; Bem and Rinzel, 2004; Wojcik et al., 2014; Reyes et al., 2015; Doloc-Mihu and Calabrese, 2016). Most of these studies focus in the analysis of alternating regular rhythms, and only a few works address the presence of irregular spiking-bursting activity in mutually inhibitory neurons, (e.g., Varona et al., 2001a; Doloc-Mihu and Calabrese, 2011; Nagornov et al., 2016).

Using conductance-based models and realistic connection topologies inspired by the crustacean pyloric CPG, in this paper we address the study of asymmetry connectivity factors shaping CPG spiking-bursting rhythms. In particular, we show the existence of asymmetric maximal synaptic conductances and time constants that shape regularized and robust alternating spiking-bursting activity in half-center oscillator circuits, and we assess their role in the rhythm configuration. We also discuss the modulation of the regularity by additional gap-junction connections to the half-center oscillator. Finally, we show how a closed-loop protocol can adapt online the synaptic time constants based on the regularity of the burst periods. We argue that this closed-loop interaction is an effective methodology to characterize the coordination properties that arise both from the connection topology and the individual dynamics of the spiking-bursting neurons in these circuits when there is no time to explore the whole parameter space, as in experimental approaches.

## 2. Materials and methods

### 2.1. Neuron model

For our analysis we use a conductance based model proposed by Komendantov and Kononenko (1996), which describes the conductances of eight membrane currents. The basic membrane potential equation is:

(1) $$\begin{array}{l} -C_{m}dV/dt=I_{Na(TTX)}+I_{K(TEA)}+I_{K}+I_{Na}+I_{Na}(V)\\ \;+\;I_{B}+I_{Ca}+I_{Ca\;-\;Ca}. \end{array}$$

The slow-wave generating mechanism is given by sodium, potassium and chemosensitive currents:

(2) $$I_{Na}(V)=g_{Na}^{*}(V)(1/(1+exp(-0.2(V+45))))(V-V_{Na});$$

(3) $$I_{Na}=g_{Na}^{*}(V-V_{Na});$$

(4) $$I_{K}=g_{K}^{*}(V-V_{K});$$

(5) $$I_{B}=g_{B}^{*}m_{B}h_{B}(V-V_{B});$$

(6) $$dm_{B}/dt=(1/(1+exp(0.4(V+34)))-m_{B})/0.05;$$

(7) $$dh_{B}/dt=(1/(1+exp(-0.55(V+43)))-h_{B})/1.5;$$

The spike-generating mechanism is described by TTX-sensitive sodium and TEA-sensitive potassium Hodgkin-Huxley type currents:

(8) $$I_{Na(TTX)}=g_{Na(TTX)}^{*}m^{3}h(V-V_{Na});$$

(9) $$I_{K(TEA)}=g_{K(TEA)}^{*}n^{4}(V-V_{K});$$

(10) dm/dt=(1/(1+exp(−0.4(V+31)))−m)/0.0005;

(11) dh/dt=(1/(1+exp(0.25(V+45)))−h)/0.01;

(12) dn/dt=(1/(1+exp(−0.18(V+25)))−n)/0.015;

The calcium transient voltage-dependent current is described by:

(13) $$I_{Ca}=g_{Ca}^{*}m_{Ca}^{2}(V-V_{Ca});$$

(14) $$dm_{Ca}/dt=(1/(1+exp(-0.2V))-m_{Ca})/0.01;$$

The calcium stationary [*Ca*^2+^]_*in*_ inhibited current is given by:

(15) $$\begin{array}{l} I_{Ca\;-\;Ca}=g_{Ca\;-\;Ca}^{*}\frac{1}{1+exp(-0.06(V+45))}\\ \;\frac{1}{1+exp(K_{\beta }([Ca]-\beta ))}(V-V_{Ca}); \end{array}$$

(16) $$d[Ca]/dt=\rho -I_{Ca}/2Fv-K_{s}[Ca];$$

where *v* = 4π*R*^3^/3 is the volume of the cell; [*Ca*] is ${\left[Ca^{2+}\right]}_{in}$ (mM), *F* is Faraday number (*F* = 96,485 *Cmol*^−1^), *K*_*s*_ is the intracellular Ca-uptake rate constant and ρ is the endogenous Ca buffer capacity.

**Figure 1:**
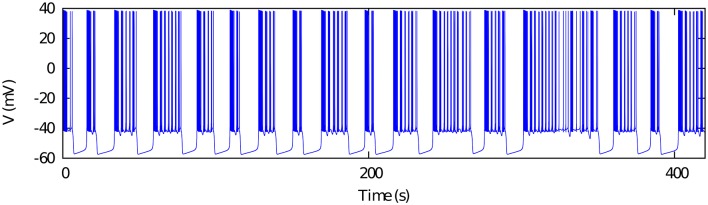
**Chaotic bursting activity of a single Komendantov-Kononenko model neuron**. Model parameters are specified in Table 1 (see also Komendantov and Kononenko, 1996). *V*^0^ = −47.0.

This Hodgkin-Huxley type model displays the dynamical richness observed in the spiking-bursting activity of several neuron types (e.g., see Komarov et al., 2008; Latorre et al., 2013) which underlies some of their rhythm negotiating properties. The parameters used in our simulations are set for the chaotic bursting regime (see Table 1). Figure 1 illustrates the irregular bursting activity of the model in this regime, which resembles the observed behavior in isolated CPG neurons (Abarbanel et al., 1996; Elson et al., 1998).

**Table 1:** **Parameters for the chaotic bursting regime used in our simulations, (see also appendix in Komendantov and Kononenko, 1996)**.

***V*_*Na*_(*mV*)**	***V*_*K*_(*mV*)**	***V*_*B*_(*mV*)**	***V*_*Ca*_(*mV*)**	***C*_*m*_*μF***	***R*(*mm*)**	***K*_*s*_(1/*s*)**	**ρ**	***K*_β_(1/*mM*)**
40	−70	−58	150	0.02	0.1	50	0.002	15000
β(mM)	$g_{K}^{*}(\mu S)$	$g_{Na}^{*}\left(\mu S\right)$	$g_{Na}^{*}\left(V\right)\left(\mu S\right)$	$g_{B}^{*}\left(\mu S\right)$	$g_{Na\left(TTX\right)}^{*}\left(\mu S\right)$	$g_{K\left(TEA\right)}^{*}\left(\mu S\right)$	$g_{Ca}^{*}\left(\mu S\right)$	$g_{Ca-Ca}^{*}(\mu S)$
0.00004	0.25	0.02	0.13	0.18	400	10	1	0.01

### 2.2. Network topologies

Synaptic asymmetries both in strength and duration are known to be present in the mutual inhibition between CPG neurons (Marder and Eisen, 1984). For our study on the asymmetry factors shaping regular and irregular bursting rhythms, we will use three different topologies of mutually inhibitory neurons that we describe below.

#### 2.2.1. Mutually inhibitory oscillator circuit with symmetric synaptic temporal dynamics

**Figure 2:**
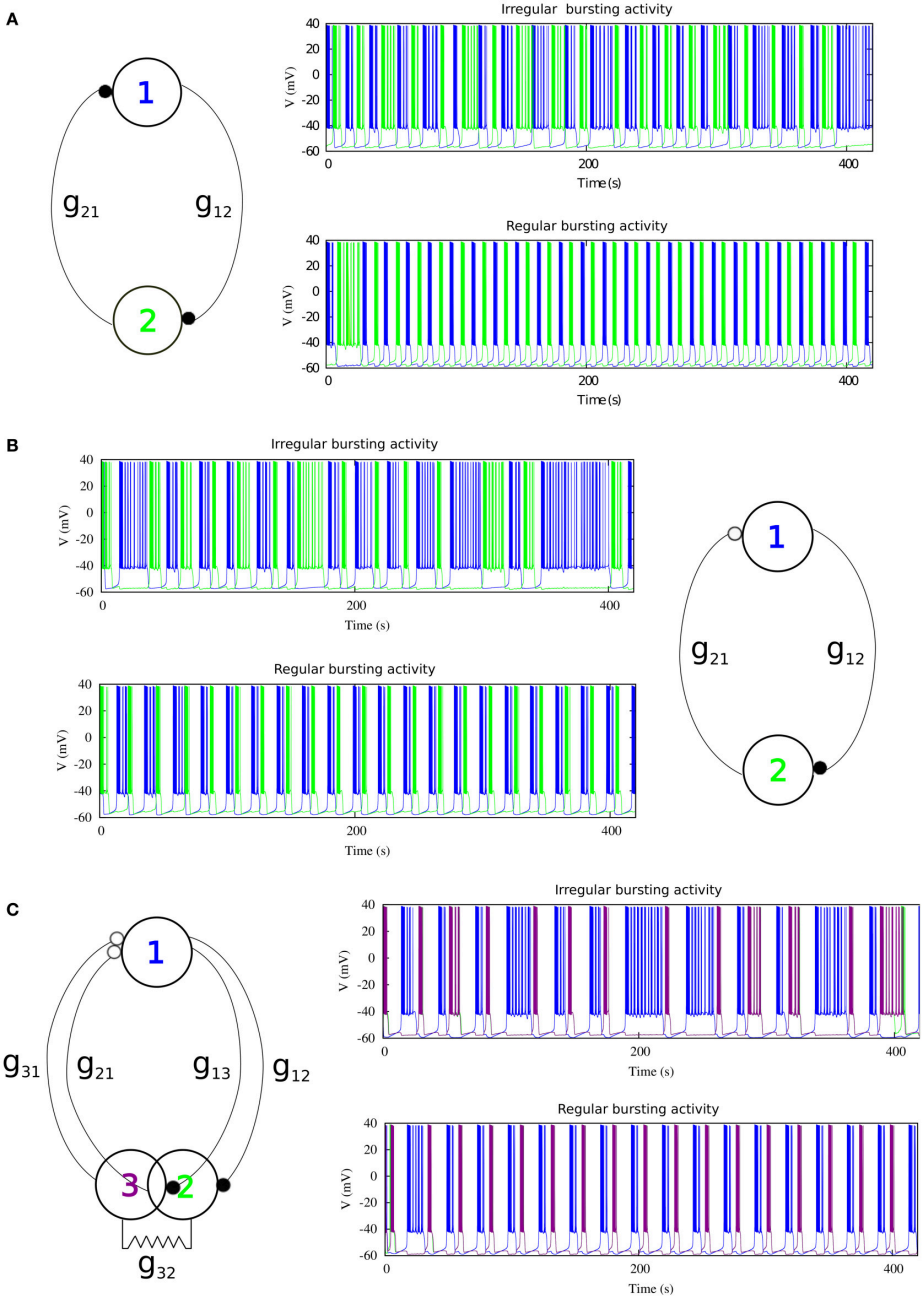
**The three different topologies of mutually inhibitory spiking-bursting neural circuits considered in this study**. The activity can be irregular or regular depending on the maximal conductances of the synapses and the rest of parameters that control the asymmetry level in each circuit. **(A)** mutually inhibitory oscillator circuit with temporal symmetric synapses. Black filled circles represent fast graded chemical synapses, see Equation (17). Parameters: Top panel *g*_21_ = 0.005 μ*S*, *g*_12_ = 0.044 μ*S* (irregular rhythm); Bottom panel *g*_21_ = 0.022 μ*S*, *g*_12_ = 0.024 μ*S* (regular rhythm). **(B)** Mutually inhibitory oscillator circuit with temporal asymmetric synapses. The empty circle represents a slow chemical synapse, see Equations (18–19). Parameters: *k*_1_ = 0.6 *ms*^−1^, *k*_2_ = 0.27 *ms*^−1^, Top panel *g*_21_ = 0.0155 μ*S*, *g*_12_ = 0.0255 μ*S*; Bottom panel *g*_21_ = 0.0235 μ*S*, *g*_12_ = 0.0055 μ*S*. **(C)** Asymmetric topology with gap-junctions modeled with Eq. (20). Parameters: *k*_1_ = 0.6 *ms*^−1^, *k*_2_ = 0.27 *ms*^−1^, Top panel *g*_21, 31_ = 0.05 μ*S*, *g*_12, 13_ = 0.03 μ*S*; Bottom panel *g*_21, 31_ = 0.035 μ*S*, *g*_12, 13_ = 0.055 μ*S*.

We first consider a half-center oscillator topology, i.e., a minimal network built up with two model neurons connected with mutually inhibitory chemical synapses that have symmetric temporal characteristics but considering the possibility of different maximal conductances in each synapse (see left panel in Figure 2A). The associated synaptic currents have been modeled with a fast graded synapse, a common type of chemical synapse in many CPGs, as follows (Golowasch et al., 1999; Latorre et al., 2002, 2006).

(17) $$I_{post}^{f}=\frac{g_{prepost}^{f}\;(V_{post}-E_{Syn})}{1.0+exp(s^{f}(V^{f}-V_{pre}))},$$

where $g_{prepost}^{f}$ is the maximal synaptic conductance of the postsynaptic neuron, one of our control parameters. *V*_*post*_ is the membrane potential of the postsynaptic neuron, *E*_*Syn*_ is the synaptic reversal potential, *V*^*f*^ determines the threshold of the graded synapse, and *V*_*pre*_ is the membrane potential of the presynaptic neuron. The values of the synapse parameters used in our simulations are *E*_*Syn*_ = −65*mV*, *V*^*f*^ = −49*mV*, *s*^*f*^ = 0.31*mV*^−1^. The mutually inhibitory connections in the circuit lead the neurons to a rhythm negotiation in the form of alternating bursting activity. The activity produced by the circuit can be regular or irregular depending on the value of the maximal conductances of the synapses as it can be observed in the right panels of Figure 2A. In this paper we have considered that the activity is regular if the coefficient of variation of the period *C*_*v*_ during five consecutive bursts is <5%.

#### 2.2.2. Mutually inhibitory oscillator circuit with asymmetric synaptic temporal dynamics

Temporal asymmetry is present between the LP and the PD neurons in the crustacean pyloric CPG (Marder and Eisen, 1984). For a further characterization of the role of asymmetry shaping CPG spiking-bursting rhythms, one of the *fast* graded inhibitory synapses in the previous circuit is replaced by a *slow* graded inhibitory synapse (see right panel in Figure 2B). The slow synaptic current is given in our model by Golowasch et al. (1999) and Latorre et al. (2002, 2006).

(18) $$I_{post}^{s}=g_{prepost}^{s}m_{post}^{s}(V_{post}-E_{Syn}),$$

where

(19) $$\frac{dm_{post}^{s}}{dt}=\frac{k_{1}(1.0-m_{post}^{s})}{1.0+exp(s^{s}(V^{s}-V_{pre}))}-k_{2}m_{post}^{s}$$

Here $g_{prepost}^{s}$ is the maximal synaptic conductance of the postsynaptic neuron, *k*_1_ and *k*_2_ are time constants which control the speed and duration of the synaptic current, and *V*^*s*^ determines the threshold of the graded slow synapse. We used *V*^*s*^ = −49 mV and *S*^*s*^ = 1.0 mV^−1^. Below we will use *k*_1_ and *k*_2_ as additional control parameters to introduce asymmetry in the temporal evolution of the synaptic currents. Examples of regular and irregular alternating bursting activity generated by this type of circuit are shown in the left panels of Figure 2B.

#### 2.2.3. Topology with gap-junction induced asymmetry

In addition to the asymmetric inhibitory connectivity in the network, we considered a third circuit in which neuron 2 of the previous circuit is replaced by two electrically coupled cells, as it is illustrated in the left panel of Figure 2C. This topology with electrical coupling is also present in several CPGs and in particular in the crustacean stomatogastric ganglion (Selverston and Moulins, 1987). The equation used to model the gap junction was:

(20) $$I_{post}^{gj}=g_{prepost}^{gj}(V_{post}-V_{pre}),$$

where the value for the gap-junction conductance used was $g_{prepost}^{gj}=0.0045mS$. Note that this third topology involves two slow inhibitory graded synapses to neuron 1 (see Figure 2C), and one fast graded inhibitory synapse to each of the electrically coupled cells (neurons 2 and 3). Again, the values of the maximal synaptic conductances determine the regular or irregular rhythm in the circuit (see right panels in Figure 2C).

## 3. Results

### 3.1. Regularized activity in mutually inhibitory oscillator circuits

**Figure 3:**
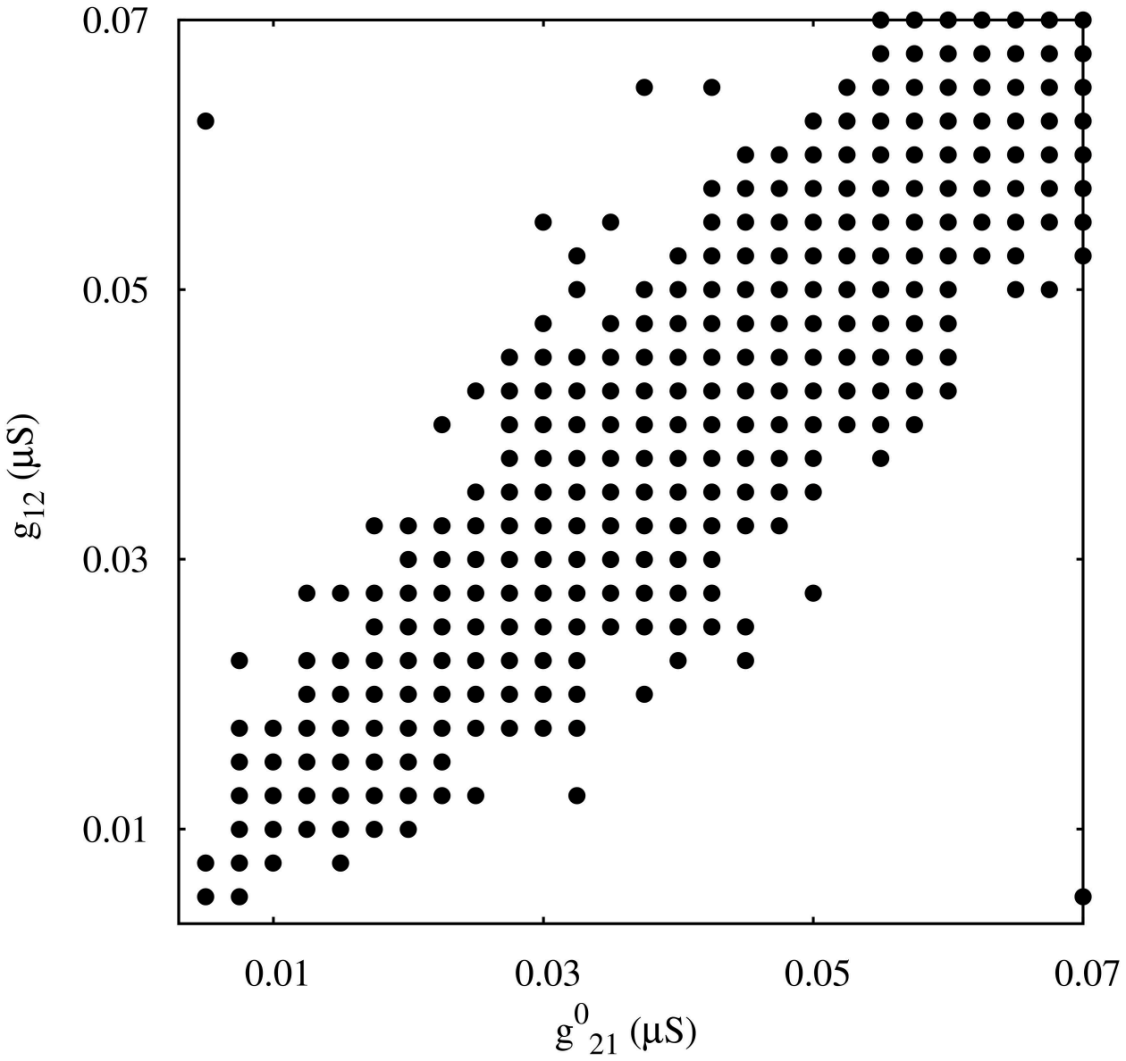
**Map of conductances *g*_12_ and *g*_21_ that lead to regularization of the spiking-bursting activity for the connection topology with fast symmetric temporal dynamics**. The incremental step for both *g*_12_ and *g*_21_ is 0.0025 μ*S*. Black circles represent values that lead to regular activity (*C*_*v*_ < 5%). The symmetric distribution of the circles in the map reflects the balanced temporal dynamics of the fast synapses, which in general allows only moderate differences in the maximal conductances to achieve regularity.

As a departing point, we have considered a half-center oscillator topology with symmetric fast temporal dynamics in the synapse model (see left panel in Figure 2A). This temporal symmetric circuit generates regular and irregular alternating bursting activity depending on the values of synaptic maximal conductances. Simulations were run to explore the synaptic maximal conductances that resulted in regular or irregular activity according to the criterion of having the coefficient of variation *C*_*v*_ of this activity below a 5% threshold. Figure 3 illustrates the map of conductances *g*_12_ and *g*_21_ that lead to regularized rhythms (black dots) consisting of alternating bursting between the two neurons. White spaces represent regions were irregular spiking-bursting activity exists. One can observe that this map is nearly symmetric, i.e., regions that lead to regularized spiking-bursting rhythms correspond to values of maximal conductances *g*_12_ and *g*_21_ that do not differ much beyond 0.02 μ*S*. Thus, for temporal symmetric fast inhibitory synapses, regularization occurs for closely symmetric maximal conductance values.

The right panel in Figure 2 shows two examples of regular and irregular alternating bursting activity for representative values of *g*_12_ and *g*_21_ conductances. Note the symmetric phase relationships of the regular spiking bursting rhythm, which corresponds to the nearly symmetric values of the maximal conductances.

### 3.2. Regularized activity in mutually inhibitory oscillator circuits with temporal asymmetry

**Figure 4:**
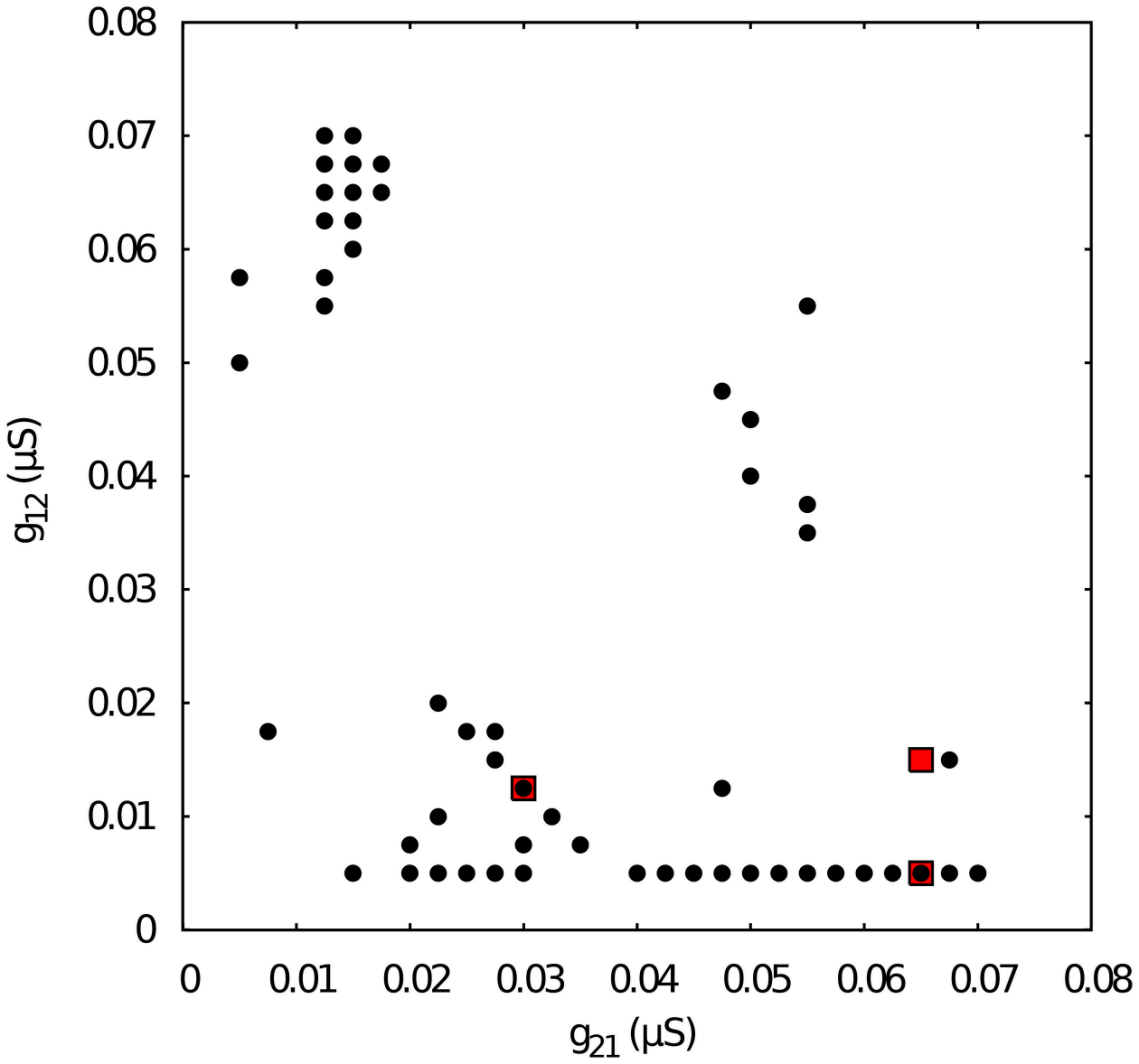
**Map of conductances *g*_12_ and *g*_21_ of the temporal asymmetric mutually inhibitory circuit that lead to regularization of the spiking-bursting activity**. The incremental step for both *g*_12_ and *g*_21_ is 0.0025 μ*S*. Black circles represent values that lead to regular activity (*C*_*v*_ < 5%). The map shows a reduced number of configurations of parameters that leads to regular activity as a consequence of the temporal asymmetry in the connection between the neurons. Parameters: *k*_1_ = 0.6 *ms*^−1^, *k*_2_ = 0.27 *ms*^−1^. Red squares correspond to specific values of *g*_12_ and *g*_21_ that will be used for subsequent analysis of *k*_1_ vs *k*_2_ maps.

Here we focus on the analysis of a mutually inhibitory circuit with temporal synaptic asymmetry. In this case, we consider mutual inhibition with a slow synapse in one direction and a fast synapse in the other (see left panel in Figure 2B). This asymmetry is present in half center oscillators that built up CPGs (Marder and Eisen, 1984). Figure 4 represents the map of conductances *g*_12_ and *g*_21_ that lead to regularized rhythms consisting of alternating spiking-bursting activity between the two neurons. It is important to emphasize that in this case asymmetry in the maximal conductances coexists with the temporal asymmetry of the slow and fast synapses in the circuit. Note the reduced size and the sparser distribution of maximal conductances that lead to regularized spiking-bursting rhythms, which is caused by the synaptic imbalance introduced by the temporal asymmetry. Figure 2B illustrates two examples of regular and irregular spiking-bursting activity, respectively, for representative values of *g*_12_ and *g*_21_ conductances. One can see the asymmetric phases of the regular rhythm as compared to the one depicted in Figure 2A.

**Figure 5:**
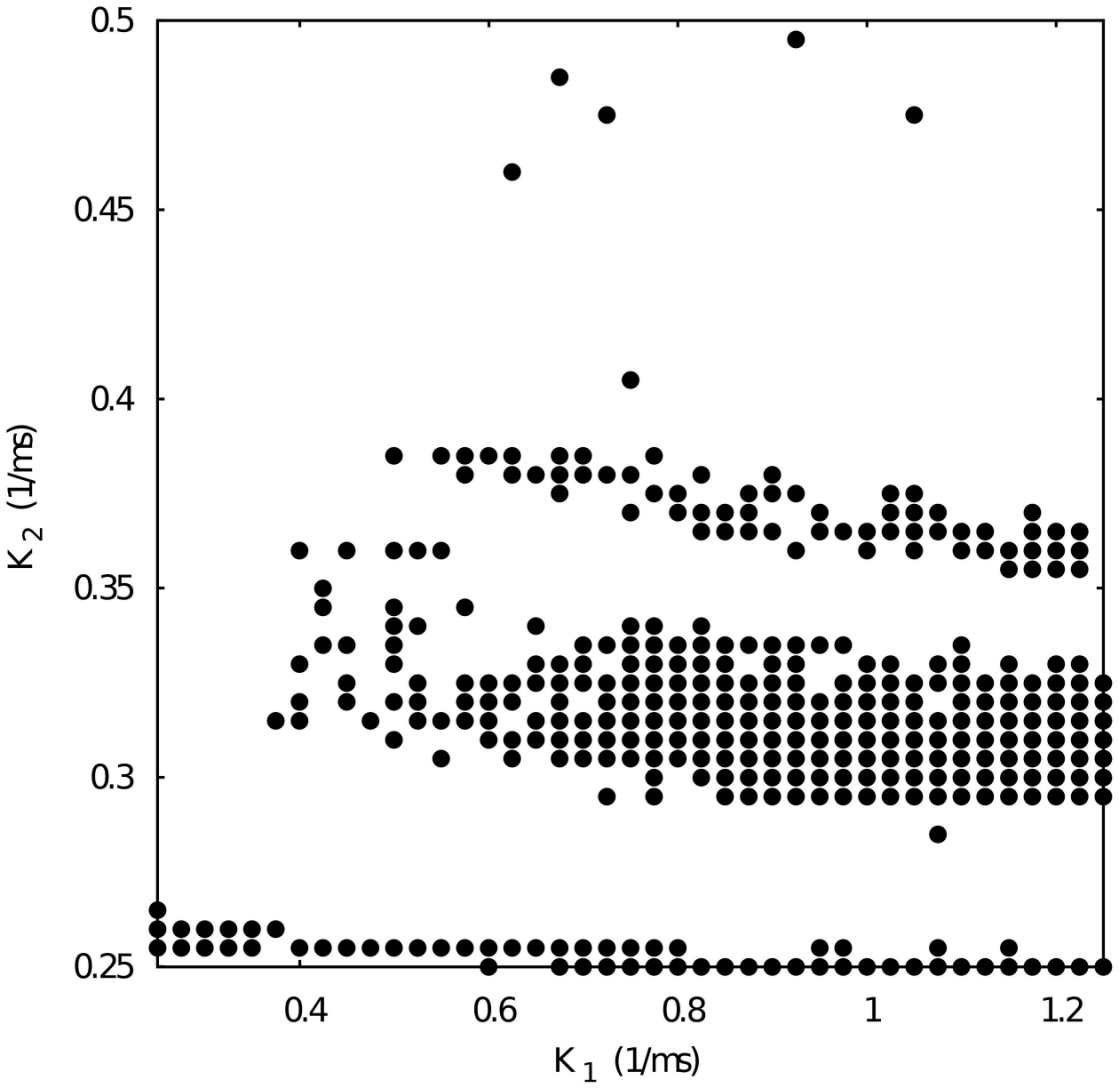
**Map of slow synaptic time constants *k*_1_ and *k*_2_ of the temporal asymmetric mutually inhibitory oscillator circuit which lead to regularization of the spiking-bursting activity**. Parameters: *g*_21_ = 0.03 μ*S*, *g*_12_ = 0.0125 μ*S* (see Figure 4 red squares). The incremental step for *k*_1_ and *k*_2_ is 0.025 *ms*^−1^ and 0.005 *ms*^−1^, respectively. Black circles represent values that lead to regular activity (*C*_*v*_ < 5%).

Next, we chose representative values of the maximal conductances in the temporal asymmetric circuit (indicated by red squares in Figure 4) and, using the same methodology, we explored the parameter space of the time constants *k*_1_ and *k*_2_, which control the temporal aspects of the slow synapse. Figure 5 shows this analysis for fixed *g*_21_ = 0.03μ*S* and *g*_12_ = 0.0125 μ*S*, corresponding to a regularized activity regime in Figure 4. Note the presence of *k*_2_ bands where regularity occurs for large regions of *k*_1_ values for this particular selection of maximal conductances. The size of these bands, and thus the size of the regions with regular activity, depend on the values of the maximal conductances of the mutually inhibitory circuit.

**Figure 6:**
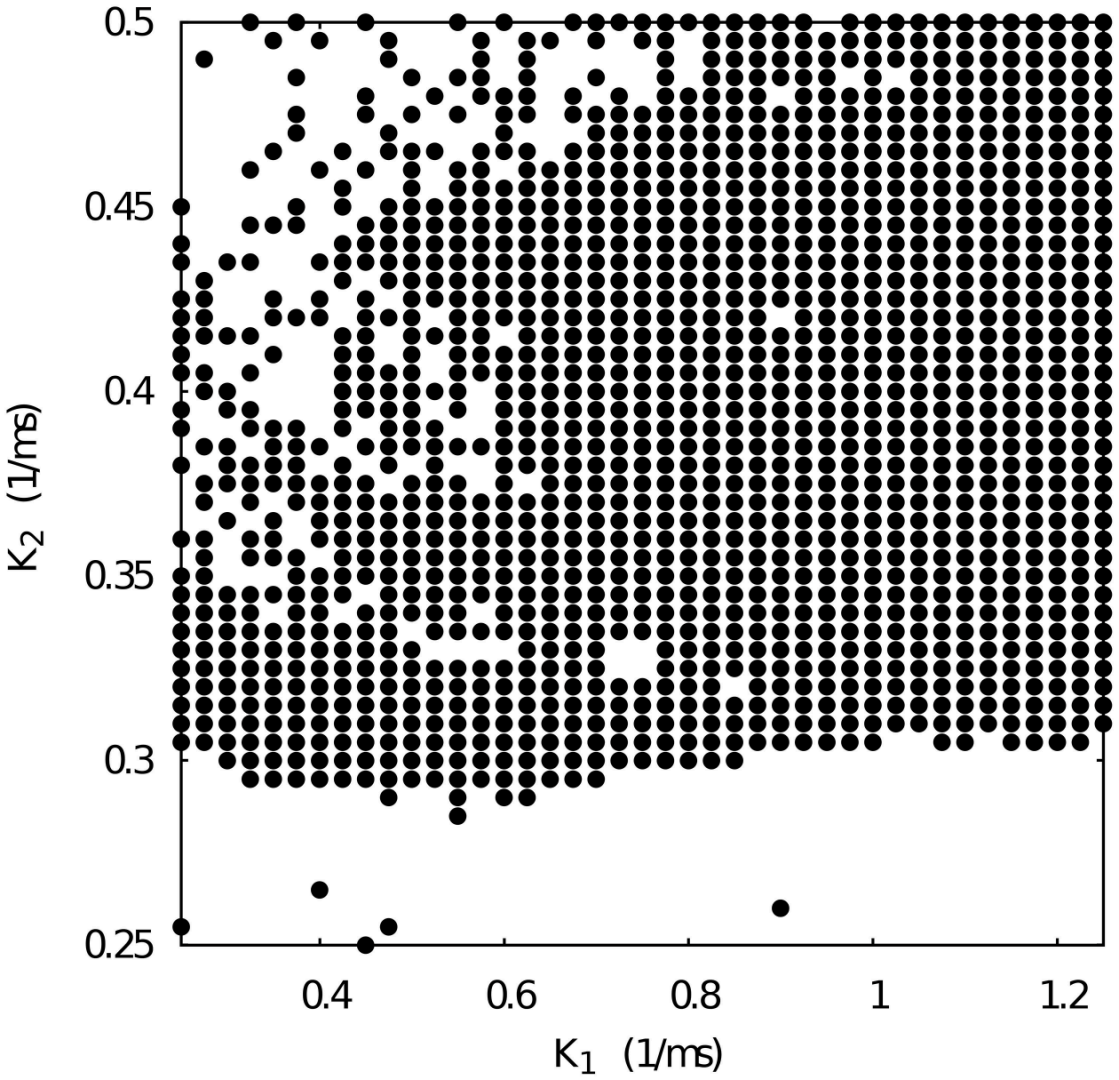
**Map of slow synaptic time constants *k*_1_ and *k*_2_ of the temporal asymmetric mutually inhibitory oscillator circuit which lead to regularization of the spiking-bursting activity**. Parameters: *g*_21_ = 0.065 μ*S*, *g*_12_ = 0.005 μ*S* (see Figure 4 red squares). The incremental step for *k*_1_ and *k*_2_ is 0.025 *ms*^−1^ and 0.005 *ms*^−1^, respectively. Black circles represent values that lead to regular activity (*C*_*v*_ < 5%).

Figure 6 shows the *k*_1_ vs *k*_2_ map for fixed *g*_21_ = 0.065 and *g*_12_ = 0.005 μ*S*. Note that such conductance values also correspond to regular spiking-bursting activity in the map depicted in Figure 4. In spite of the large maximal conductance difference, the resulting combination of conductance and temporal asymmetries in the mutual inhibition lead to a broad region of regular spiking-bursting activity. Irregular regimes are mostly in the region defined by *k*_2_ < 0.3

**Figure 7:**
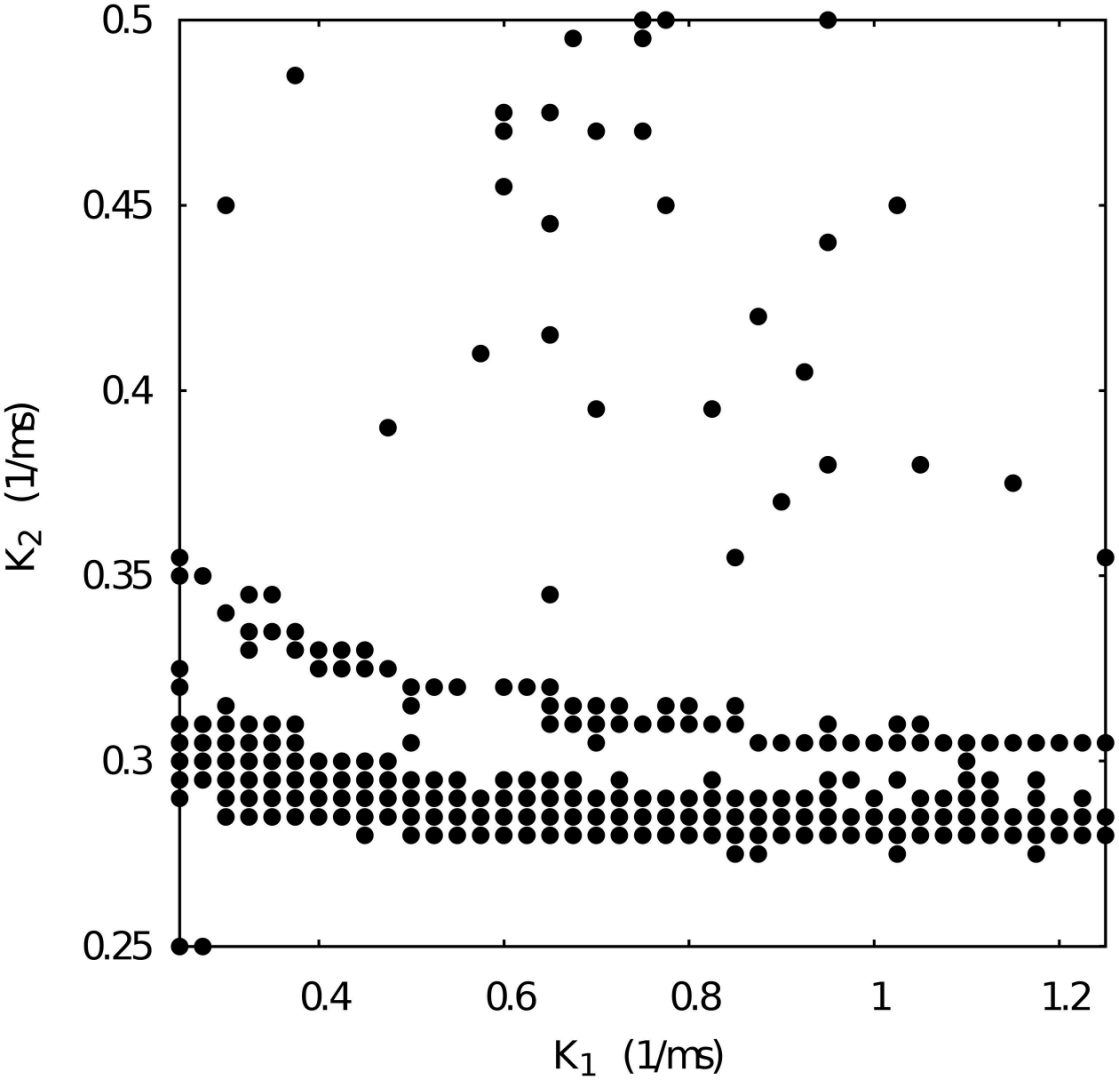
**Map of slow synaptic time constants *k*_1_ and *k*_2_ of the temporal asymmetric mutually inhibitory oscillator circuit which lead to regularization of the spiking-bursting activity**. Parameters: *g*_21_ = 0.065 μ*S*, *g*_12_ = 0.015 μ*S* (see Figure 4 red squares). The incremental step for *k*_1_ and *k*_2_ is 0.025 *ms*^−1^ and 0.005 *ms*^−1^, respectively. Black circles represent values that lead to regular activity (*C*_*v*_ < 5%).

When we select from Figure 4 a set of maximal conductances that correspond to irregular spiking-bursting activity (e.g., *g*_21_ = 0.065 and *g*_12_ = 0.015 μ*S*), we obtain a *k*_1_ vs *k*_2_ map with reduced regularity bands (see Figure 7). Nevertheless, one can observe that the temporal asymmetry can compensate the conductance unbalance to achieve regular regimes.

### 3.3. Regularized activity in mutually inhibitory oscillator circuits with gap-junction induced asymmetry

Finally, in addition to the asymmetric connectivity in the network, we have considered another source of asymmetry common in many CPG's (Selverston and Moulins, 1987) by replacing one of the neurons in the former circuit with two electrically coupled cells (see left panel in Figure 2C). The gap-junction synchronizes the activity of neurons 2 and 3. It is important to note that in this configuration there are two slow and two fast inhibitory graded synapses. Note also the phase asymmetry of the regular regimes in this configuration. To analyze the contribution of this topological asymmetry to the generation of regular and irregular rhythms, we have fixed the synaptic time constants to *k*_1_ = 0.6 *ms*^−1^, *k*_2_ = 0.27 *ms*^−1^, and the gap-junction conductance $g_{23,32}^{gj}$ = 0.0045 μ*S*.

**Figure 8:**
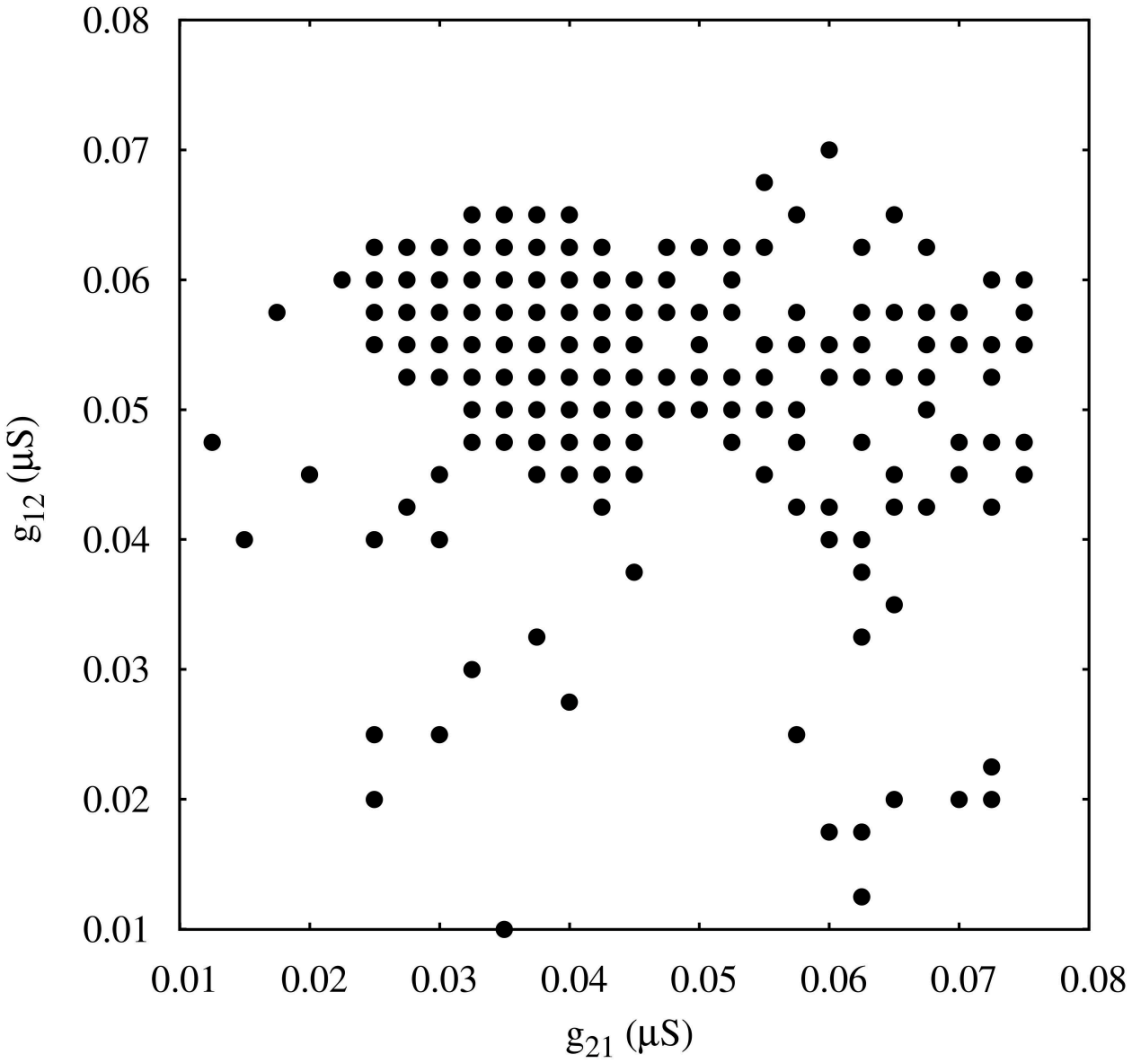
**Map of conductances *g*_12_ and *g*_21_ of the circuits with gap-junction induced asymmetry that lead to regularization of the spiking-bursting activity where the incremental step for both *g*_12_ and *g*_21_ is 0.0025 μ*S***. Black circles represent values that lead to regular activity (*C*_*v*_ < 5%). Parameters: *k*_1_ = 0.6 *ms*^−1^, *k*_2_ = 0.27 *ms*^−1^, $g_{23,32}^{gj}$ = 0.0045 μ* S*.

Figure 8 shows the map of conductances *g*_12_ and *g*_21_ that lead to regularized alternating bursting activity in this configuration. One can observe that the regularity regions are larger than in the corresponding case for the circuit with only temporal synaptic asymmetry (cf. Figure 4). This result hints that the gap-junction connectivity, with its associated dynamical inertia, could contribute to enlarge the regions of regular spiking-bursting activity in living half-center oscillators.

## 4. Closed-loop exploration for regular bursting rhythms

In the previous sections we have presented a modeling study in minimal mutually inhibitory circuits of the asymmetry factors that contribute to shape regular and irregular spiking-bursting rhythms. How could the hypotheses drawn in this paper be tested experimentally? Dynamic clamp can be used to modify maximal conductances and synaptic temporal characteristics by building hybrid circuits composed of living neurons and artificial synapses (Sharp et al., 1993; Destexhe and Bal, 2009). As the time to perform such experiments is restricted and the number of configurations that can be tested is typically small, we propose here a close-loop exploration method to allow such study. The closed-loop algorithm can control the dynamics by changing a set of parameters of the network to achieve a specific goal (e.g., the regularization of the bursting activity). These set of parameters are updated in every iteration according to a designed rule (which should be simple due to time restrictions) until the goal is achieved.

We validate the method in the context of the half-center oscillator model with temporal asymmetric synapses and *g*_21_ = 0.065 μ*S*, *g*_12_ = 0.005 μ*S*. In this case, the closed-loop algorithm updates the value of the synaptic time constants *k*_1, 2_ according to the difference between the current period *P*_1_ and the average of the periods of the last five bursts 〈*P*_1_〉 trying to find a suitable new set of values that lead to regular activity *C*_*v*_ < 5%, which is the goal of this closed-loop exploration. The departing points are different sets of configurations of the synaptic time constants $k_{1}^{0}$ and $k_{2}^{0}$ that drive the system into alternating irregular spiking-bursting activity. Every time a new burst *n* is generated in neuron 1, the algorithm checks if the coefficient of variation of the period calculated for the last 5 bursts *C*_*v*_ is below the established regularity threshold 5%. Then, the value of the synaptic time constants are updated as follows:

$$k_{1,2}^{n}=k_{1,2}^{n\;-\;1}-\alpha \cdot \left(\langle P_{1}\rangle -P_{1}^{n}\right)$$

where α is the rate for the synaptic time constant change, 〈*P*_1_〉 is the average period of neuron 1 calculated with the last five bursts and $P_{1}^{n}$ is the current period. In this study we set α = 0.01. This value and the monitoring of the last five bursts provide a good balance between a reasonable online measurement of the regularity and a fast convergence to the closed-loop goal.

**Figure 9:**
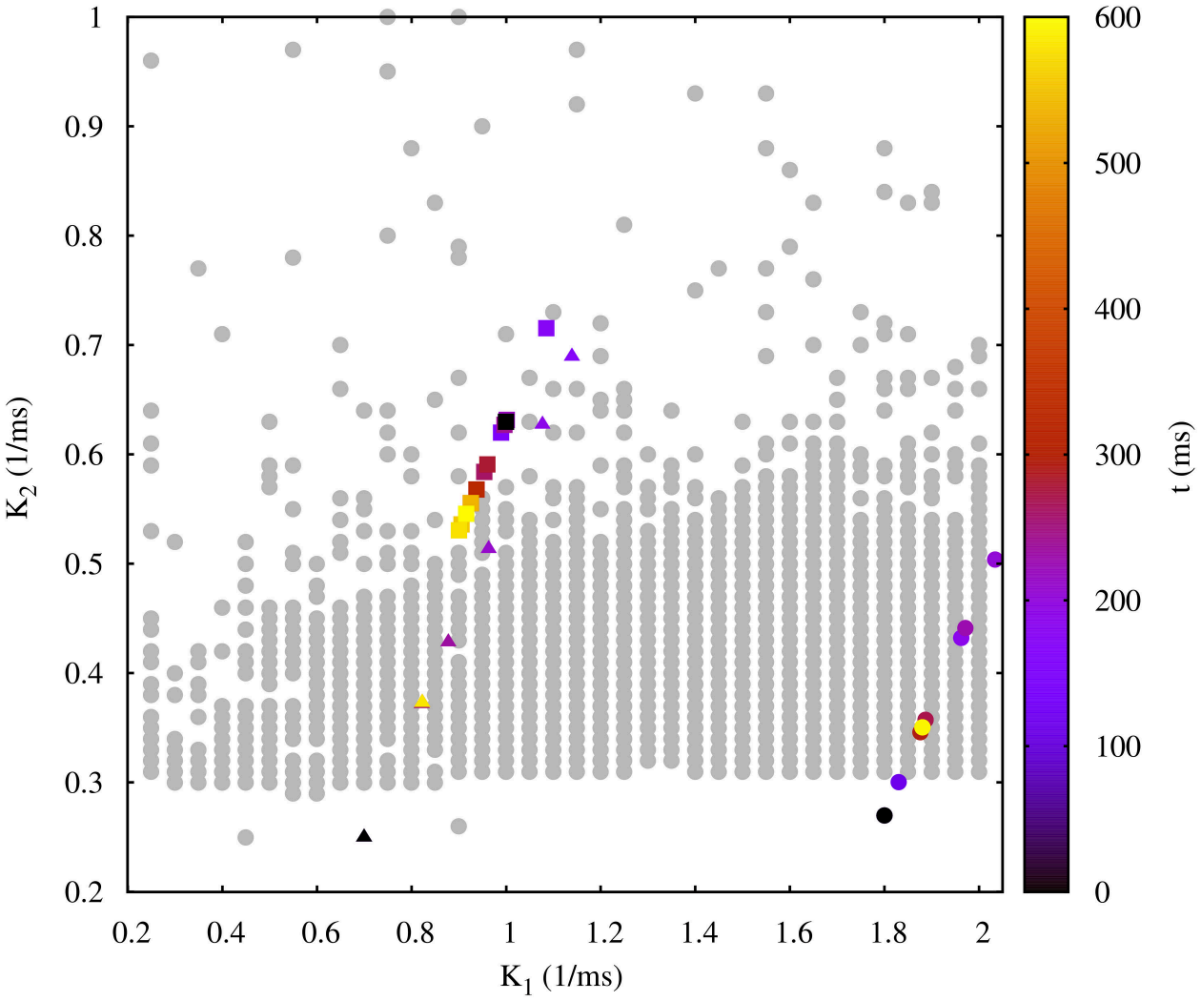
**Evolution of the synaptic time constants *k*_1_ and *k*_2_ of the low synapse in a half-center oscillator during closed-loop exploration for regular bursting rhythms**. The figure shows the evolution of the values of *k*_1_ and *k*_2_ in time (color bar in *s*) for three different departing values (coded in black): squares points, $k_{1}^{0}$ = 1.00 *ms*^−1^, $k_{2}^{0}$ = 0.63 *ms*^−1^; triangle points, $k_{1}^{0}$ = 0.70 *ms*^−1^, $k_{2}^{0}$ = 0.25 *ms*^−1^; circle points, $k_{1}^{0}$ = 1.80 *ms*^−1^, $k_{2}^{0}$ = 0.27 *ms*^−1^. Final values after the closed-loop exploration (coded in yellow): squares points, $k_{1}^{f}$ = 0.92 *ms*^−1^, $k_{2}^{f}$ = 0.55 *ms*^−1^; triangle points, $k_{1}^{f}$ = 0.82 *ms*^−1^, $k_{2}^{f}$ = 0.37 *ms*^−1^; circle points, $k_{1}^{f}$ = 1.89 *ms*^−1^, $k_{2}^{f}$ = 0.35 *ms*^−1^. Gray circles in the background represent the map of slow synaptic time constants *k*_1_ and *k*_2_ that lead to regularization of the spiking-bursting activity. This map was generated as explained in the previous sections and is depicted for the validation of the closed-loop search. The incremental step for *k*_1_ and *k*_2_ is 0.05 *ms*^−1^ and 0.01 *ms*^−1^, respectively. The protocol searchers for a suitable value of both synaptic time constants which drives the system into a regular bursting activity, *C*_*v*_ < 5% (gray circle points). Parameters: *g*_21_ = 0.065 μ*S*, *g*_12_ = 0.005 μ*S*.

**Figure 10:**
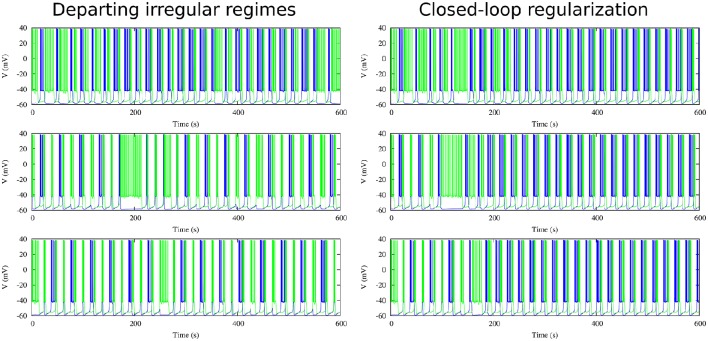
**Departing irregular regimes and closed-loop exploration for regular activity for the three cases discussed in Figure 9 in a circuit with synaptic temporal asymmetry**. Left panels show the departing irregular activity for the following slow synaptic time constants: $k_{1}^{0}$ = 1.00 *ms*^−1^, $k_{2}^{0}$ = 0.63 *ms*^−1^, $k_{1}^{0}$ = 0.70 *ms*^−1^, $k_{2}^{0}$ = 0.25 *ms*^−1^ and $k_{1}^{0}$ = 1.80 *ms*^−1^, $k_{2}^{0}$ = 0.27 *ms*^−1^. Right panels show the regularization obtained through the closed-loop (cf. evolution of time constants in Figure 9). Note that the protocol is able to find the regular regimes in a very short time. Maximal conductance parameters: *g*_21_ = 0.065 μ*S*, *g*_12_ = 0.005 μ*S*.

Figure 9 shows the evolution of three sets of departing time constants $k_{1}^{0}$ and $k_{2}^{0}$ during the closed-loop protocol displayed over a map of slow synaptic time constants that lead to regularized rhythms (gray circles). In these three examples the departing values are located in empty regions in the maps (*C*_*v*_ > 5%) and at the end of the protocol the new set of values $k_{1}^{f}$ and $k_{2}^{f}$ are located within the gray circles area (*C*_*v*_ < 5%). Noting the color code, one can observe that the changes in $k_{1,2}^{n}$ can become quite large during the first few interactions since this change is proportional to the difference between the current period and the average of the periods of the last five bursts. The protocol is able to find the regular spiking-bursting regimes in only a few iterations. The departing irregularity and the achieved regularized activity of the circuit after the closed-loop protocol are shown in Figure 10.

This close-loop protocol allows different configurations and optimizations. The experimenter can choose to modify time constants *k*_1_ and *k*_2_ simultaneously or one at a time, for example only *k*_2_ to unveil the regularity bands shown in Figures 5, 7. The protocol can also include a nonlinear dependence on the difference between the current and the averaged bursting period, as well as other search methods (e.g. gradient descent, stochastic search) and alternative performance measurements to evaluate online the regularization goal.

## 5. Discussion

Using realistic conductance-based models of half-center oscillator based CPG circuits, in this paper we have addressed the study of synaptic asymmetry to shape robust alternating spiking-bursting rhythms. In particular, we focused on the analysis of asymmetry of synaptic maximal conductances and time constants in the reciprocal inhibition of half center oscillators. We have mapped the regimes of regular and irregular coordinated rhythms as a function of these parameters. We have shown that asymmetry both in the maximal conductances and in the temporal dynamics of mutually inhibitory neurons, including modulation by gap-junction connectivity, can synergistically contribute to shape large regions of regular spiking-bursting regimes in central pattern generator circuits. Regular rhythms resulting from realistic asymmetric synaptic configurations display specific phase relationships that reflect the balance among the distinct sources of asymmetry.

Irregularity regimes are typically disregarded both in experimental and theoretical CPG research. One reason for this is that regular rhythms, as recorded in experimental setups, are more easily associated with observable rhythmic CPG motor functions. Several theoretical studies have shown that regular regimes could be more efficient for specific motor tasks (Huerta et al., 2000, 2001; Stiesberg et al., 2007). However, coordinated irregularity is also present in living CPGs under normal and pathological circumstances and might mediate key aspects of the rhythm negotiation in these circuits. Recent brute force approaches to map the parameter space in half center oscillators models inspired by the leech heartbeat CPG have also pointed out the presence of irregular regimes (Doloc-Mihu and Calabrese, 2011, 2016). In this paper, we have shown that the asymmetric synaptic parameter space for the existence of regular and irregular coordinated rhythms in a conductance-based half-center oscillator is large. Previous modeling efforts that did not explore synaptic asymmetry hinted that intrinsic neuron irregularity easily disappeared under mutual inhibition in CPG half-center oscillator circuits, (e.g., see Varona et al., 2001a,b). Here we have seen how half-center oscillations can be modulated and coordinated by asymmetrical factors in the mutual inhibition of its constituent neurons. The study of irregular regimes in the case of realistic asymmetric synapses can shed further light to understand the balance between robustness and flexibility in central pattern generator circuits.

There are known asymmetries in the connectivity of CPG half-center oscillators. However, their role in shaping the circuit rhythm has not been explored in detail. In this paper, we have shown that a closed-loop protocol adapting online the synaptic parameters under a regularization goal can be an effective methodology to map and characterize the coordination properties that arise both from the connection topology, including their asymmetry, and the individual neuronal dynamics in these circuits. We believe that customized versions of this protocol can be used in experimental setups, where there is no time to explore the whole parameter space, to address the hypotheses drawn by the discussed model.

## Author contributions

IE and PV designed the study. IE performed the simulations. IE and PV analyzed the data and wrote the paper. All authors approved the final version for publication.

## Funding

We acknowledge support from MINECO/FEDER DPI2015-65833-P and TIN-2012-30883 (http://www.mineco.gob.es/) and ONRG grant N62909-14-1-N279.

### Conflict of interest statement

The authors declare that the research was conducted in the absence of any commercial or financial relationships that could be construed as a potential conflict of interest.
